# Evidence of cochlear neural degeneration in normal-hearing subjects with tinnitus

**DOI:** 10.1038/s41598-023-46741-5

**Published:** 2023-11-30

**Authors:** Viacheslav Vasilkov, Benjamin Caswell-Midwinter, Yan Zhao, Victor de Gruttola, David H. Jung, M. Charles Liberman, Stéphane F. Maison

**Affiliations:** 1 Eaton-Peabody Laboratories, Massachusetts Eye and Ear, 243 Charles Street, Boston, MA 02114 USA; 2 Department of Otolaryngology, Harvard Medical School, Boston, MA 02114 USA; 3 Department of Biostatistics, Harvard T.H. Chan School of Public Health, Boston, MA 02114 USA

**Keywords:** Auditory system, Cochlea, Inner ear

## Abstract

Tinnitus, reduced sound-level tolerance, and difficulties hearing in noisy environments are the most common complaints associated with sensorineural hearing loss in adult populations. This study aims to clarify if cochlear neural degeneration estimated in a large pool of participants with normal audiograms is associated with self-report of tinnitus using a test battery probing the different stages of the auditory processing from hair cell responses to the auditory reflexes of the brainstem. Self-report of chronic tinnitus was significantly associated with (1) reduced cochlear nerve responses, (2) weaker middle-ear muscle reflexes, (3) stronger medial olivocochlear efferent reflexes and (4) hyperactivity in the central auditory pathways. These results support the model of tinnitus generation whereby decreased neural activity from a damaged cochlea can elicit hyperactivity from decreased inhibition in the central nervous system.

## Introduction

An estimated 10–15% of the adult population worldwide experiences tinnitus^1,2^. The tinnitus percept becomes debilitating in 2–4% of the population, causing sleep deprivation, social isolation, anxiety and depression, adversely affecting work performance, and resulting in a severe decline in the quality of life^3^. Most therapeutic interventions, including sound maskers^4^, tinnitus-retraining therapy^5^, and other cognitive behavioral therapies^6^, are primarily designed to alleviate the distress caused by the tinnitus percept. At this time, there is no cure, and silencing tinnitus will remain elusive until its biological origins are established.

Along with difficulties understanding speech in noisy environments, tinnitus is one of the most common complaints of patients with sensorineural hearing loss (SNHL). A common model of tinnitus generation postulates that decreased neural activity from a damaged cochlea can elicit hyperactivity from decreased inhibition in the central nervous system. In animal models of SNHL, the loss of synaptic connection to inner hair cells leaves many surviving spiral ganglion neurons without any spontaneous activity or response to sound^7^. In contrast, central auditory circuits often show increased spontaneous and/or sound-evoked firing, that can be associated with behavioral signs of tinnitus^8–10^ and hyperacusis^11,12^. This is hypothesized to arise from a maladaptive neural compensation, with hyperactivity due to decreased inhibition in the central auditory pathways^12–18^ in response to a loss of peripheral input^13,19–23^.

The discovery that permanent damage to the cochlear nerve can arise after acoustic overexposure and during aging, even when the sensory cells remain intact^24,25^, reconciled this model with the existence of tinnitus in patients with normal audiometric sensitivity^26,27^. Indeed, this cochlear neural degeneration (CND) does not elevate thresholds until it becomes extreme^28,29^, in part because the most vulnerable neurons are those with high thresholds and low spontaneous rates (SRs)^30,31^ that do not contribute to threshold detection in quiet^32^.

Tinnitus is common in hearing-loss etiologies in which the underlying pathology is likely to include massive CND^33^, e.g. in patients with Ménière’s disease, vestibular schwannoma, neurofibromatosis of type II and sudden SNHL^34–39^. However, attempts to demonstrate an association between CND and tinnitus in those with normal thresholds have produced mixed results. In two studies of young participants, tinnitus was associated with speech intelligibility deficits^40^ or with an estimate of noise exposure history^41^, two variables closely associated with CND as shown in animal^42,43^ and human studies^33,44–46^. However, no evidence of peripheral neural deficits was found, as assessed via measures of the suprathreshold amplitudes of wave I obtained from Auditory Brainstem Responses (ABRs). These results contrast with several reports showing reduced ABR wave I amplitudes in tinnitus patients audiometrically matched to controls^47–51^. In the latter studies, the observed peripheral neural deficit was associated with an increased central gain, as interpreted from the amplitude of ABR wave V. Additional humans studies have linked tinnitus to other metrics thought to assess CND including the middle-ear muscle reflex (MEMR)^52^ or the ratio between the summating potential (SP) and the cochlear nerve response (action potential [AP]) as measured via electrocochleography^53^.

Possible reasons for this discrepancy include (1) the large inter-subject variability in ABR amplitudes^54,55^ and tinnitus percept^17^, (2) the analysis protocols used to extract the cochlear nerve responses from ABR waveforms^56–58^, (3) the stimulus parameters including level, repetition rate and final spectrum reaching the ear, and (4) thresholds at extended high frequencies (EHFs), which are not assayed by standard audiometry but can respond to stimuli at moderate and high SPLs and thus contribute to auditory evoked potentials. Besides these technical differences, group comparisons are complicated by the likelihood that some control subjects have cochlear damage that does not cause tinnitus, as central changes must underlie the development of a phantom percept^16^.

Another way to gain insight into CND and possible changes in auditory central gain is to assay the feedback reflexes to the auditory periphery, i.e. the MEMR or the medial olivocochlear efferent reflex (MOCR). Although loss of afferent signal due to CND should impact the effector neurons of these reflexes, particularly if low-SR afferent fibers are over-represented in their ascending inputs^59,60^, increasing central gain could have opposing effects. In one study of normal-hearing humans, subjects with tinnitus showed increased MOCR strength^61^, while in another, MEMR strength was reduced in those with tinnitus^52^.

To further probe a possible association between CND and tinnitus, we recruited a large cohort of normal-hearing participants with minimal loss at EHFs (≤ 20 dB HL) and extracted, under computer control, peripheral and central markers from both auditory evoked potentials and auditory efferent reflexes.

## Materials and methods

This study was approved by the Institutional Review Board of the Massachusetts General Brigham. All aspects were conducted in accordance with the relevant regulations of the institution. Recruitment was undertaken irrespective of the participant’s tinnitus status.

### Inclusion criteria

All participants were native speakers of English, in good health, between the ages of 18 and 72, with no history of ear or hearing problems including no history of somatic/objective tinnitus as defined by AAO-HNS guidelines^62^ (e.g., pulsatile/whooshing sounds pulsating in synchrony with heartbeat^63^, or caused by temporo-mandibular joint dysfunction^64^). At the time of testing, all participants had unremarkable otoscopic examinations and normal middle-ear function as assessed via the Titan Suite from Interacoustics, with a probe-tone frequency of 226 Hz and an ear-canal pressure change ranging from − 300 daPa to + 200 daPa in each ear, to ensure that ear canal volume, tympanic membrane mobility and middle-ear pressure were normal. There were no additional inclusion criteria beyond the ability to give voluntary informed written consent.

### Subject pool and grouping

Three groups of participants were defined based on self-report: (1) those who never experienced tinnitus or occasionally heard phantom sounds that emerged and resolved within minutes (control group), (2) those who experienced at least one episode of temporary/intermittent tinnitus^62,65^ of less than six months duration, or (3) those who reported a continuous tinnitus percept for more than 6 months^62^. All participants reporting tinnitus completed a questionnaire describing their tinnitus percept, including lateralization and degree of spectral complexity^66^. All questionnaires were completed in a quiet room (not a sound booth) before any testing to ensure that the rating of tinnitus percept was not affected by auditory stimulation.

### Audiometric thresholds

As described in previous studies^44,67,68^: audiometric thresholds were obtained using Interacoustics Equinox 4.0 with the High Hz option. Pure-tone air-conduction (AC) thresholds were measured at standard audiometric frequencies from 0.25 to 8 kHz, plus 3 and 6 kHz, using DD45 headphones. To minimize changes in sound levels due to standing waves and improve intra-subject reliability of threshold estimates above 8 kHz, we measured AC thresholds at extended high-frequencies using warble-tones delivered via a circumaural HDA200 high-frequency headset. Only participants with normal thresholds (≤ 20 dB HL) and mean audiometric thresholds at EHFs (measured at 9, 10, 11.2, 12.5, 14 and 16 kHz) below or equal to 20 dB HL were included in this study.

### Auditory brainstem responses/electrocochleography

Subjects’ ear canals were prepped by scrubbing with a cotton swab coated in Nuprep®. Electrode gel was applied on the cleaned portion of the canal and over the gold-foil of ER3- 26A/B tiptrodes before insertion. A horizontal montage was used, with a ground on the forehead at midline, one tiptrode as the inverting electrode and the other as the non-inverting electrode in the opposite ear. Low (< 5 kΩ) and balanced impedance readings were obtained with inter-electrode impedance values within 2 kΩ of each other. Stimuli were generated by our custom rig and stimulus waveforms were transduced and delivered via silicone tubing connected to ER-3A insert earphones. Stimuli were 100 µs-clicks delivered at either 125 or 110 dB pSPL in alternating polarity at a rate of 9.1 or 40.1 Hz in the presence or absence of a 90-ms forward masker (8–16 kHz, 5-ms ramp) terminating 6 ms before the click onset. The spectrum of the masking noise at the output of the ER-3A are described in Grant et al. (2020)^44^. The total noise dose for all ECochG measurements was well within OSHA and NIOSH standards. Data acquisition was handled by the Interacoustics Eclipse hardware and software. Electrical responses were amplified 100,000×, and 2000 sweeps were averaged for each recording. Average traces acquired by the Eclipse software (passband [3.3–5000 Hz]) were exported to Matlab for further analyses using custom scripts. Specifically, ECochG waveforms were processed as described in Vasilkov et al.^69^ through two Infinite Impulse Response (IRR) filters with a steepness of 0.95 and a stopband attenuation of 60 dB to separate the contributions of auditory-nerve spikes from other generators^69^. The cutoff frequencies were [3.3–470 Hz] for the low-pass filter and [470–3000 Hz] for the bandpass filter.

### Middle-ear muscle reflex

As described in Mepani et al.^68^, stimulus generation and data acquisition were controlled by our custom rig based on 24-bit digital input–output boards from National Instruments in a PXI chassis, with custom software control via LabVIEW. Response and stimulus waveforms, to and from the input–output boards, were transduced via microphone and dual sound sources in an ER-10X system (Etymotics Research). Changes in ear-canal sound pressure to a click probe were evoked by an ipsilateral noise elicitor. Specifically, we use a pair of 100-μs clicks at 95 dB pSPL separated by a 500-ms elicitor (white noise burst with a 2.5 ms ramp) presented 30 ms after the first click and preceding the second by 5 ms. This click-noise-click complex was repeated every 2035 ms, leaving 1.5 s of silence between noise bursts to allow relaxation of the MEMs. Four complexes were presented at each elicitor level, and elicitor level was raised in 5 dB steps from 40 to 95 dB SPL. To eliminate click-evoked otoacoustic emissions, the waveforms were truncated at 2 ms after the peak of the click. For each ear, the entire process was repeated three times and averaged. For each average, the spectral difference (gain) between the two click waveforms was computed.

Threshold was defined as the lowest elicitor level at which the gain emerged from the noise floor by 1 standard deviation in the following conditions: (1) within a 1,000 Hz wide band where the largest magnitude of the ear canal SPL was recorded; (2) within a 1,000 Hz wide band where the largest gain was recorded across the 500–5000 Hz window; (3) within a 1,000 Hz band centered on the frequency where the lowest threshold was recorded across the 500–5000 Hz window; (4) across the summed gains within the 500–2000 Hz window; and (5) across the summed gains within the 500–5000 Hz window. To compute MEMR strength, the absolute values of the gain were summed across the above bands/windows for an elicitor level of 95 dB SPL.

### Medial olivocochlear reflex

Transient-evoked otoacoustic emissions (TEOAEs) were measured in each ear in response to 500 sweeps of a 4-click complex (32.5 ms inter-click interval) in non-linear mode, where the first 3 clicks were presented at 65 dB peak SPL and the fourth was 9.5 dB higher and inverted in polarity. The summed response, i.e., the non-linear component, was windowed to include times from 4 to 23 ms after the peak of the click response, high-pass filtered from 750 Hz and Fourier transformed to produce the spectrum of the TEOAEs. Responses were compared with vs. without a contralateral elicitor consisting of a continuous broadband noise presented prior initiating the ipsilateral click train. The medial olivocochlear reflex (MOCR) was measured as the average difference between the TEOAE spectra in the frequency band between 1 and 2.8 kHz, as suggested by a detailed comparison of different techniques for measuring MOCR^70^. To be included, each TEAOE must be at least 5 dB above the noise floor and present at each measured frequency band (1–2.8 kHz).

### Statistical analyses

Inter-group age differences were assessed using a one-way ANOVA. Chi-squared tests were used to assess equality of proportions across groups. The binary outcomes considered were sex, concussion, anxiety/depression, difficulties hearing in noise and occupational/recreational noise exposure).

Audiometric threshold differences were analyzed using linear regression to evaluate the group effect across frequencies. The intermittent-tinnitus group’s threshold was defined as reference category, to which both *no tinnitus* and *chronic tinnitus* groups were compared.

To investigate the joint effect of predictor variables on outcomes, mixed-effects multivariable regression models were fit, with a random intercept for each participant. These models allow for the presence of correlation between outcome measures that are done independently on each ear. Predictor variables included mean threshold at standard audiometric frequencies, mean thresholds at EHFs, sex, history of concussion and tinnitus status. Threshold and sex were selected as predictor variables because of their previously reported association with the variance of auditory evoked-potentials^54,71^. We selected threshold rather than age, because age and threshold are highly correlated^68^ and there is evidence of age-related neural deficits in normal-hearing subjects as measured via ABRs^72,73^ or in histopathological studies of human temporal bones^45,74^. Outcomes that were calculated as ratios (N_1_^*^/N_2_^*^, N_1_^*^/N_3_^*^ and N_1_^*^/N_5_^*^) were log transformed to make their distributions more symmetric. The rare negative values of these quantities were assigned the value of 0.01.

A permutation test was conducted to test whether the lowpass waveforms differed in amplitude across groups within the first 6 ms. The test was performed by randomly permuting group labels 10,000 times and recording the average difference in amplitude for each permutation. Two-sided p-values were obtained by comparing each test statistic with the associated permutation distribution.

## Results

We sought to determine if the inferred CND of individuals with normal audiograms was correlated with their self-report of chronic tinnitus using a test battery probing the different stages of the auditory processing from the hair cell responses of the inner ear to the auditory reflexes of the brainstem.

**Figure 1:**
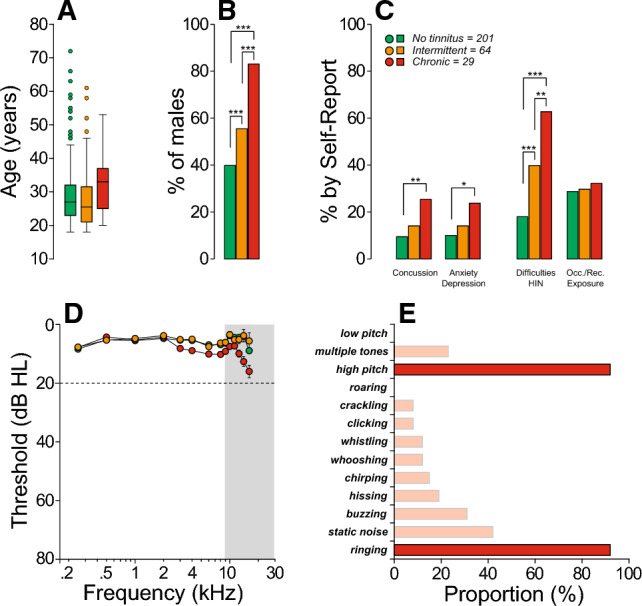
Patient characteristics. (**A**) Box and whisker plots of participant ages, grouped based on tinnitus status. (**B**) Sex distribution for each group of participants. (**C**) Survey results obtained from the medical history and questionnaires. (**D**) Audiometric thresholds at standard and extended high frequency (EHFs; grey box) for each group. Dotted line at 20 dB HL separates normal hearing from hearing loss, as defined in clinical settings. (**E**) Survey results from participants reporting tinnitus describing their tinnitus percept. Legend in (**C**) shows the number of participants in each group and applies to all panels. Significance of group differences are indicated by brackets: *p < 0.05, **p < 0.01; ***p < 0.001.

We recruited 294 subjects (140 females, 154 males), from 18 to 72 years old, with normal audiometric thresholds in both ears and with mean thresholds at EHFs (9–16 kHz) ≤ 20 dB HL (Fig. 1D). Each participant completed a series of questionnaires regarding their medical history related to ear or hearing, including a thorough tinnitus screening^75^. A total of 201 participants reported no previous experience with tinnitus (“no tinnitus” group) beyond the transient perception of a sound that emerged and resolved within a minute^76^. 64 participants had experienced a temporary/intermittent tinnitus, often associated with a recent episode of noise exposure (e.g., after attending a concert) or certain medications^77^. These participants, along with those having experienced a constant subjective tinnitus^62,65^ for less than 6 months duration were included in the “intermittent tinnitus” group. Lastly, 29 participants included in the “chronic tinnitus” group^62^ were experiencing a constant subjective tinnitus for at least 6 months. All but one participant from the latter group reported tinnitus bilaterally.

As shown in Fig. 1A, there were no significant age differences across groups (one-way ANOVA, p = 0.507); however, sex differences were highly significant (Chi-squared tests, p < 0.001): while a majority of participants from the *no tinnitus* group were female (~ 57%), ~ 85% of those with *chronic tinnitus* were male (Fig. 1B). Those with chronic tinnitus reported a previous concussion, anxiety and/or depression, misophonia and difficulties hearing in noisy environments more often than those who never experienced tinnitus (Chi-squared tests, see Fig. 1C). When present, the tinnitus percept was typically described as a high-pitched ringing (Fig. 1E). Interestingly, self-report of recreational or occupational exposures to loud sounds was not different across groups (Chi-squared tests, p = 0.371). Per inclusion criteria, all participants had normal audiometric thresholds; however, as illustrated in Fig. 1D, participants with chronic tinnitus showed significantly poorer hearing sensitivity, particularly at EHFs, when compared to the no-tinnitus or intermittent-tinnitus groups (Suppl. Table S1). Threshold differences between the no-tinnitus and intermittent-tinnitus groups were not significant (Suppl. Table S1).

### Auditory brainstem responses

**Figure 2:**
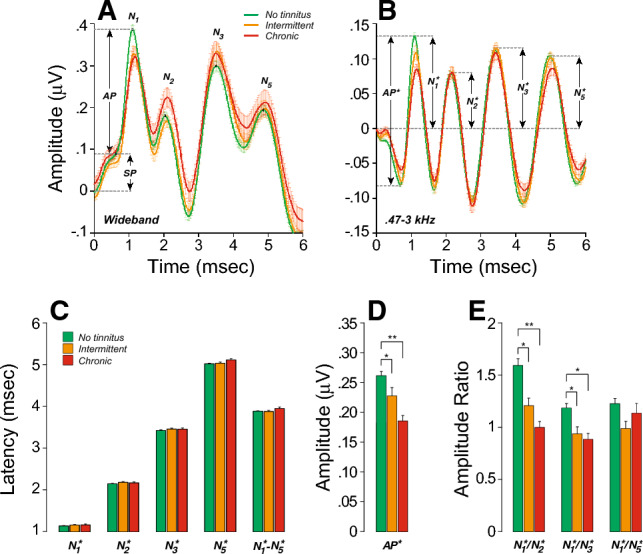
Participants with chronic tinnitus show peripheral neural deficits associated with increases in central gain. (**A**,**B**) Averaged click-evoked ECochG (± SEMs) obtained from each group. Baseline is defined as the mean pre-onset amplitude (− 2 to 0 ms). When extracted by visual inspection (**A**), the Summating Potential (SP) is defined as the difference between baseline and the last inflection point on the rising phase of the first peak after stimulus onset (1–2 ms). (N1); the Action Potential (AP) is defined as the amplitude difference between SP and N_1_. When analyzed under computer control after band-pass filtering (0.47–3 kHz), AP^*^ is defined as the trough-to-peak amplitude of the first wave. Legend in **A** also applies to (**B**). (**C–E**) Measures of mean (± SEM) latencies (**C**), amplitudes (**D**) and amplitude ratios (**E**) as extracted from individual filtered waveforms and averaged for each group. Legend in (**C**) also applies to (**D**) and (**E**). Significance of group differences after adjusting for EHFs is indicated: *p < 0.05, **p < 0.01; ***p < 0.001.

To probe the relationship between tinnitus and CND, we measured auditory-evoked potentials from each participant via ABRs/electrocochleography (ECochG). As illustrated in Fig. 2A, the early responses of ECochG waveforms include both the summating potential (SP), a mixture of pre- and post-synaptic analog potentials, and the action potential (AP), the summation of all-or-nothing spikes from the auditory nerve. As noted in previous studies^44,58,68,69^, it is important to differentiate Wave I from AP, as the AP rides on top of the summating potential (SP) “pedestal” that arises from multiple generators of different polarities^56,57^ (sensory cells, non-spiking and spiking neural components). Likewise, measuring N_1_P_1_ is suboptimal, because the P_1_ includes the repolarization phase of short-latency auditory-nerve spikes that can be cancelled by the depolarization phases of longer latency spikes from more apical locations, as well as by early spikes from the cochlear nucleus. As recently described^69^, we use high-pass filtering method (Fig. 2B) to separate the neural spiking components from other cellular generators and to identify, under computer control, each EcochG marker, defining AP^*^ as the trough-to-peak amplitude *within* the first 1.5 ms as a measure of the cochlear nerve response. Given that tinnitus has been linked to hyperactivity in central auditory pathways^8,78^, we also analyzed the amplitude ratios, latencies, and inter-peak latencies of the later waves II, III, and V (also known as N_2_, N_3_ and N_5_).

As described in “Materials and methods”, a mixed-effects regression analysis was used to determine the joint effect of thresholds (at standard or extended high frequencies), sex, history of concussion and tinnitus status on EcochG variables. As shown in Table 1, EHF thresholds and chronic tinnitus were significant predictors of AP^*^ amplitude, including when concussion and sex were added as predictors and when interactions between sex and groups were considered. In other words, chronic tinnitus remains a significant predictor of AP^*^ amplitude even when differences in thresholds, sex and past history of concussion are accounted for. Pairwise comparisons further show that patients with chronic or intermittent tinnitus had significantly smaller AP^*^ amplitudes and greater N_2_^*^/N_1_^*^ and N_3_^*^/N_1_^*^ amplitude ratios when compared to the no tinnitus group (Suppl. Table S2, Fig. 2). These results are consistent with peripheral neural deficits and increased central activity in “normal-hearing” participants with chronic tinnitus.

**Table 1:** Mixed-effects regression analysis with AP^*^ amplitude as outcome variable.

Predictors	Est	CI	p
(Intercept)	0.171	0.148 to 0.195	** < 0.001**
Standard	0.000	− 0.002 to 0.003	0.945
EHFs	− 0.002	− 0.002 to − 0.001	** < 0.001**
Concussion	− 0.011	− 0.038 to 0.017	0.438
Sex	0.020	− 0.002 to 0.043	0.078
Chronic	− 0.042	− 0.078 to − 0.006	**0.021**
Intermittent	− 0.005	− 0.037 to 0.028	0.782
Sex × chronic	0.058	− 0.010 to 0.127	0.095
Sex × intermittent	− 0.020	− 0.067 to 0.028	0.418
Random effects			
σ^2^	0.00		
τ_∞_	0.00_ID_		
ICC	0.66		
N	199_ID_		
Observations	387		
Marginal R^2^	0.138		
Conditional R^2^	0.711		

*Chronic* chronic tinnitus group, *CI* confidence interval, *Cond.* Conditional, *EHFs* thresholds at extended high frequencies, *Est.* estimates, *Intermittent* intermittent tinnitus group, *Marg.* marginal.

Significant values are in bold.

Interestingly, patients who reported intermittent tinnitus had AP^*^ amplitudes, as well as N_2_^*^/N_1_^*^ and N_3_^*^/N_1_^*^ ratios, that were intermediate between no-tinnitus controls and chronic tinnitus (Fig. 2D,E). However, mixed-effects regression did not show a significant predictive power of the intermittent tinnitus status on AP^*^ amplitude (Table 1). This result is not surprising, given the limited tinnitus durations in this group. Thus, in the analysis that follows, we will only compare no-tinnitus controls to those with chronic tinnitus.

### Effects of forward masking and rate of stimulus presentation

**Figure 3:**
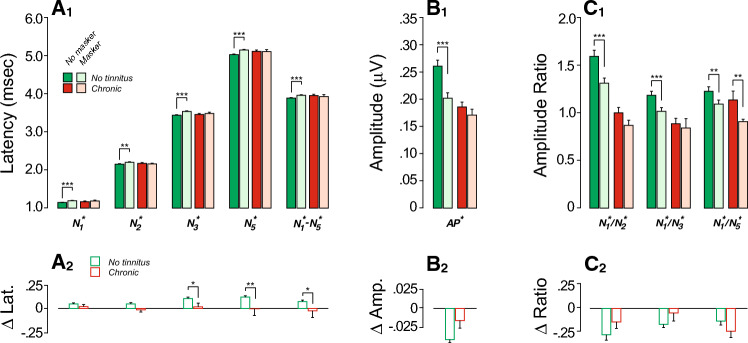
Masking effects on ECochG markers are reduced in those with chronic tinnitus. (**A**_**1**_–**C**_**1**_) Mean ABR peak latencies (**A**), amplitudes (**B**) or amplitude ratios (**C**) obtained in the absence or with a forward masker (± SEMs). Significance of paired comparisons is indicated by brackets: *p < 0.05, **p < 0.01, ***p < 0.001. (**A**_**2**_–**C**_**2**_) Masker effect adjusted for EHFs. Significance of group differences after adjusting for EHFs is indicated by brackets: *p < 0.05, **p < 0.01.

The click-evoked responses were obtained in presence or in absence of a forward masker devised to explore the contribution of EHFs (Fig. 3). The masker was set at 25 dB above masker threshold, as assessed behaviorally in each individual. Presenting of a forward masker should decrease the neural component of the response (e.g., AP^*^) without affecting the hair cell responses. Pairwise comparisons showed that, indeed, controls had significantly smaller AP^*^ amplitudes when the masker was present; an effect that was interestingly absent in the chronic tinnitus group (Suppl. Table S3, Fig. 3B_1_). However, masker-induced amplitude reductions were not statistically significant between groups (Fig. 3B_2_, Table 2).

**Table 2:** Mixed-effects regression models of masker effect as a function of tinnitus status and EHFs on ABR variables.

Predictors	On AP* amp			On log (N_1_*/N_2_*)			On log (N_1_*/N_3_*)		
	Est	CI	p	Est	CI	p	Est	CI	p
(Intercept)	− 0.039	− 0.050 to − 0.028	** < 0.001**	− 0.230	− 0.339 to − 0.120	** < 0.001**	− 0.211	− 0.316 to − 0.106	** < 0.001**
Chronic	0.020	− 0.006 to 0.047	0.135	0.008	− 0.253 to 0.268	0.955	− 0.058	− 0.308 to 0.192	0.650
EHFs	0.000	− 0.001 to 0.001	0.521	0.008	− 0.000 to 0.016	0.054	0.010	0.002 to 0.017	**0.011**
Random effects									
σ^2^	0.00			0.30			0.21		
τ_∞_	0.00_ID_			0.17_ID_			0.19_ID_		
ICC	0.42			0.36			0.47		
N	158_ID_			158_ID_			158_ID_		
Observations	298			298			298		
Marginal R^2^	0.014			0.015			0.027		
Conditional R^2^	0.424			0.371			0.487		

Predictors	On log(N_1_*/N_5_*)			On N_1_* lat			On N_2_* lat		
	Est	CI	p	Est	CI	p	Est	CI	p
(Intercept)	− 0.138	− 0.244 to − 0.031	**0.012**	0.039	0.014 to 0.063	**0.002**	0.030	− 0.003 to 0.064	0.079
Chronic	− 0.181	− 0.436 to 0.073	0.162	− 0.018	− 0.077 to 0.040	0.535	− 0.056	− 0.137 to 0.024	0.170
EHFs	0.007	− 0.000 to 0.015	0.056	0.001	− 0.001 to 0.003	0.286	0.003	0.000 to 0.005	**0.022**
Random effects									
σ^2^	0.19			0.01			0.02		
τ_∞_	0.21_ID_			0.01_ID_			0.02_ID_		
ICC	0.53			0.38			0.50		
N	158_ID_			158_ID_			158_ID_		
Observations	298			298			298		
Marginal R^2^	0.022			0.006			0.027		
Conditional R^2^	0.538			0.379			0.510		

Predictors	On N_3_* lat			On N_5_* lat			On N_1_*–N_5_* lat		
	Est	CI	p	Est	CI	p	Est	CI	p
(Intercept)	0.082	0.047 to 0.117	** < 0.001**	0.093	0.053 to 0.134	** < 0.001**	0.052	0.008 to 0.095	**0.019**
Chronic	− 0.101	− 0.183 to − 0.018	**0.017**	− 0.133	− 0.229 to − 0.036	**0.007**	− 0.115	− 0.219 to − 0.011	**0.030**
EHFs	0.003	0.000 to 0.005	**0.036**	0.002	− 0.001 to 0.005	0.111	0.002	− 0.001 to 0.005	0.283
Random effects									
σ^2^	0.03			0.03			0.03		
τ_∞_	0.02_ID_			0.03_ID_			0.04_ID_		
ICC	0.37			0.43			0.54		
N	158_ID_			158_ID_			158_ID_		
Observations	298			298			298		
Marginal R^2^	0.037			0.039			0.026		
Conditional R^2^	0.392			0.457			0.551		

*Adj*. adjusted, *Chronic* chronic tinnitus group, *CI* confidence interval, *EHFs* thresholds at extended high frequencies, *Est.* estimates.

Significant values are in bold.

Given prior reports suggesting that reductions in masker-evoked latency shifts are a marker of CND^79^, we also considered the effect of masking on response latencies. Here, the masking-evoked delays in N_1_, N_2_, N_3_ and N_5_ latencies and prolongation of the N_1_-N_5_ inter-peak latency seen in controls (Suppl. Table S3, Fig. 3A_1_) were absent in the chronic tinnitus group. Inter-group comparisons of masking effects were significant for N_3_ and N_5_ latencies, and for N_1_^*^-N_5_^*^ inter-peak latency, even after adjusting for EHFs (Fig. 3A_2_, Table 2).

**Figure 4:**
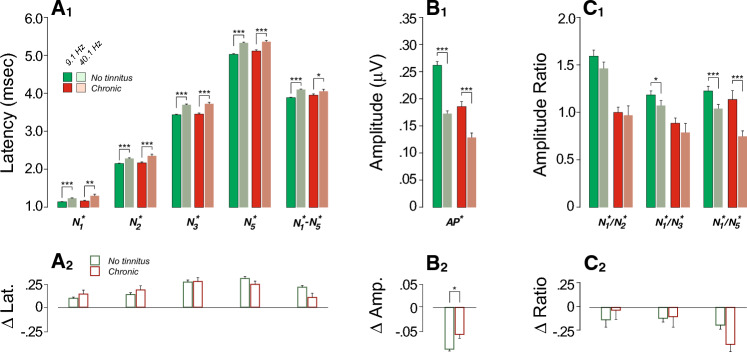
Effects of stimulus presentation rate on ECochG markers are reduced in those with chronic tinnitus. (**A**_**1**_**–C**_**1**_) Mean ABR peak latencies (**A**), amplitudes (**B**) or amplitude ratios (**C**) evoked by clicks delivered at 9.1 Hz vs. 40.1 Hz (± SEMs). Significance of paired comparisons is indicated by brackets: *p < 0.05, **p < 0.01, ***p < 0.001. (**A**_**2**_**–C**_**2**_) Click rate effects. Significance of group differences after adjusting for EHFs is indicated by brackets: *p < 0.05.

To further explore the robustness of cochlear neural responses, we assessed their fatigability by increasing the click rate. As expected, increasing the presentation rate from 9.1 Hz to 40.1 Hz led to longer peak latencies and N_1_-N_5_ interpeak latency (Fig. 4A_1_) and smaller AP^*^ amplitudes (Fig. 4B_1_) in both groups (Suppl. Table S4, Fig. 4). However, those with chronic tinnitus had significantly smaller effects on AP^*^ amplitude (Fig. 4B_2_), even after adjusting for EHFs (Table 3).

**Table 3:** Mixed-effects regression models of rate effect as a function of tinnitus status and EHFs on ABR variables.

Predictors	On AP* amp			On log (N_1_*/N_2_*)			On log (N_1_*/N_3_*)		
	Est	CI	p	Est	CI	p	Est	CI	p
(Intercept)	− 0.095	− 0.105 to − 0.085	** < 0.001**	− 0.109	− 0.255 to 0.037	0.141	− 0.196	− 0.323 to − 0.068	**0.003**
Chronic	0.026	0.002 to 0.050	**0.033**	0.013	− 0.341 to 0.367	0.943	− 0.081	− 0.390 to 0.228	0.608
EHFs	0.001	0.000 to 0.002	**0.009**	− 0.004	− 0.014 to 0.006	0.449	0.003	− 0.006 to 0.013	0.481
Random effects									
σ^2^	0.00			0.34			0.32		
τ_∞_	0.00_ID_			0.42_ID_			0.29_ID_		
ICC	0.48			0.56			0.48		
N	160_ID_			160_ID_			160_ID_		
Observations	301			301			301		
Marginal R^2^	0.055			0.002			0.002		
Conditional R^2^	0.508			0.559			0.477		

Predictors	On log(N_1_*/N_5_*)			On N_1_* lat			On N_2_* lat		
	Est	CI	p	Est	CI	p	Est	CI	p
(Intercept)	− 0.207	− 0.339 to − 0.076	**0.002**	0.098	0.068 to 0.128	** < 0.001**	0.131	0.078 to 0.185	** < 0.001**
Chronic	− 0.218	− 0.537 to 0.101	0.180	0.040	− 0.032 to 0.112	0.273	0.016	− 0.113 to 0.144	0.812
EHFs	0.000	− 0.009 to 0.009	0.960	− 0.000	− 0.003 to 0.002	0.729	0.002	− 0.002 to 0.006	0.371
Random effects									
σ^2^	0.24			0.05			0.05		
τ_∞_	0.36_ID_			0.00_ID_			0.05_ID_		
ICC	0.61			0.02			0.51		
N	160_ID_			160_ID_			160_ID_		
Observations	301			301			301		
Marginal R^2^	0.009			0.004			0.004		
Conditional R^2^	0.609			0.022			0.516		

Predictors	On N_3_* lat			On N_5_* lat			On N_1_*–N_5_* lat		
	Est	CI	p	Est	CI	p	Est	CI	p
(Intercept)	0.256	0.196 to 0.316	** < 0.001**	0.298	0.237 to 0.360	** < 0.001**	0.199	0.152 to 0.246	** < 0.001**
Chronic	− 0.008	− 0.152 to 0.136	0.912	− 0.070	− 0.219 to 0.079	0.357	− 0.109	− 0.221 to 0.004	0.058
EHFs	0.002	− 0.002 to 0.006	0.323	0.001	− 0.003 to 0.006	0.543	0.002	− 0.002 to 0.005	0.355
Random effects									
σ^2^	0.07			0.07			0.07		
τ_∞_	0.06_ID_			0.07_ID_			0.03_ID_		
ICC	0.48			0.52			0.27		
N	160_ID_			160_ID_			160_ID_		
Observations	301			301			301		
Marginal R^2^	0.004			0.005			0.017		
Conditional R^2^	0.487			0.524			0.280		

*Adj.* adjusted, *Chronic* chronic tinnitus group, *CI* confidence interval, *EHFs* thresholds at extended high frequencies, *Est.* estimates.

Significant values are in bold.

### Effects of stimulus presentation level

**Figure 5:**
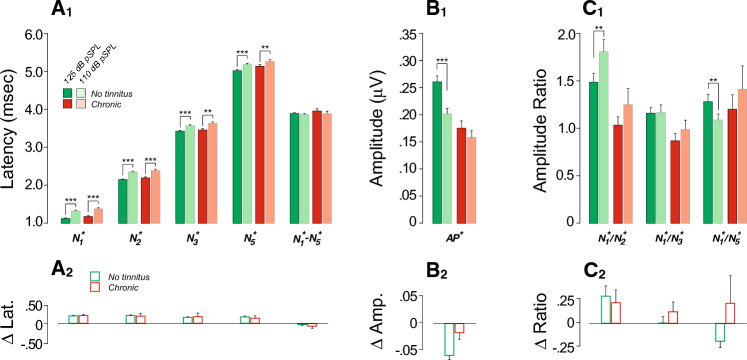
ECochG markers of chronic tinnitus are stimulus-level dependent. (**A**_**1**_–**C**_**1**_) Mean ABR peak latencies (**A**), amplitudes (**B**) or amplitude ratios (**C**) evoked by clicks delivered at 125 dB pSPL vs. 110 dB pSPL (± SEMs). Significance of paired comparisons is indicated by brackets: *p < 0.05, **p < 0.01, ***p < 0.001. (**A**_**2**_**–C**_**2**_) Stimulus level effects.

To further probe the contribution of high-threshold, low-SR fibers, we measured ABR responses at two click levels: a moderate level designed to saturate the low-threshold fibers and a higher level to additionally recruit the high-threshold fibers. If CND is selective for low-SR fibers, the difference between response at the two levels should be smaller in those with CND. Indeed, only controls showed a significant level effect on AP^*^ (Fig. 5B_1_, Suppl. Table S5). However, the chronic tinnitus group had poorer EHF thresholds, which could limit spread of excitation as stimulus level increases. Indeed the intergroup differences in this stimulus-level effect didn’t reach the statistically significant level after adjusting for EHFs (p = 0.079, Fig. 5A_2_, B_2_, C_2_; Table 4).

**Table 4:** Mixed− effects regression models of stimulus level effect as a function of tinnitus status and EHFs on ABR variables.

Predictors	On AP* amp			On log (N_1_*/N_2_*)			On log (N_1_*/N_3_*)		
	Est	CI	p	Est	CI	p	Est	CI	p
(Intercept)	− 0.070	− 0.090 to − 0.050	** < 0.001**	− 0.027	− 0.300 to 0.245	0.842	− 0.211	− 0.422 to − 0.001	**0.049**
Chronic	0.038	− 0.004 to 0.080	0.079	0.078	− 0.499 to 0.655	0.790	0.214	− 0.228 to 0.655	0.340
EHFs	0.001	− 0.000 to 0.003	0.083	0.010	− 0.007 to 0.027	0.243	0.013	− 0.000 to 0.027	0.055
Random effects									
σ^2^	0.00			0.29			0.31		
τ_∞_	0.00_ID_			0.61_ID_			0.28_ID_		
ICC	0.25			0.67			0.48		
N	67_ID_			67_ID_			67_ID_		
Observations	122			122			122		
Marginal R^2^	0.061			0.015			0.050		
Conditional R^2^	0.294			0.678			0.504		

Predictors	On log(N_1_*/N_5_*)			On N_1_* lat			On N_2_* lat		
	Est	CI	p	Est	CI	p	Est	CI	p
(INTERCEPT)	− 0.258	− 0.474 to − 0.043	**0.019**	0.216	0.169 to 0.262	** < 0.001**	0.201	0.154 to 0.249	** < 0.001**
Chronic	0.187	− 0.265 to 0.638	0.415	0.025	− 0.072 to 0.122	0.615	− 0.014	− 0.113 to 0.086	0.786
EHFs	0.011	− 0.003 to 0.025	0.108	− 0.004	− 0.007 to − 0.001	**0.022**	0.000	− 0.003 to 0.003	0.929
Random effects									
σ^2^	0.32			0.04			0.03		
τ_∞_	0.29_ID_			0.00_ID_			0.01_ID_		
ICC	0.47			0.01			0.18		
N	67_ID_			67_ID_			67_ID_		
Observations	122			122			122		
Marginal R^2^	0.036			0.044			0.001		
Conditional R^2^	0.493			0.055			0.181		

Predictors	On N_3_* lat			On N_5_* lat			On N_1_*− N_5_* lat		
	Est	CI	p	Est	CI	p	Est	CI	p
(Intercept)	0.168	0.126 to 0.210	** < 0.001**	0.141	0.087 to 0.194	** < 0.001**	− 0.041	− 0.131 to 0.050	0.375
Chronic	0.002	− 0.085 to 0.090	0.956	− 0.044	− 0.155 to 0.068	0.438	− 0.036	− 0.224 to 0.152	0.704
EHFs	− 0.000	− 0.003 to 0.002	0.750	0.003	− 0.000 to 0.007	0.077	0.004	− 0.002 to 0.010	0.158
Random effects									
σ^2^	0.03			0.05			0.08		
τ_∞_	0.00_ID_			0.00_ID_			0.04_ID_		
ICC	0.10						0.32		
N	67_ID_			67_ID_			67_ID_		
Observations	122			122			122		
Marginal R^2^	0.001			0.029			0.020		
Conditional R^2^	0.101			N/A			0.331		

*Adj.* adjusted, *Chronic* chronic tinnitus group, *CI* confidence interval, *EHFs* thresholds at extended high frequencies, *Est.* estimates.

Significant values are in bold.

### Assessment of auditory efferent reflexes

**Figure 6:**
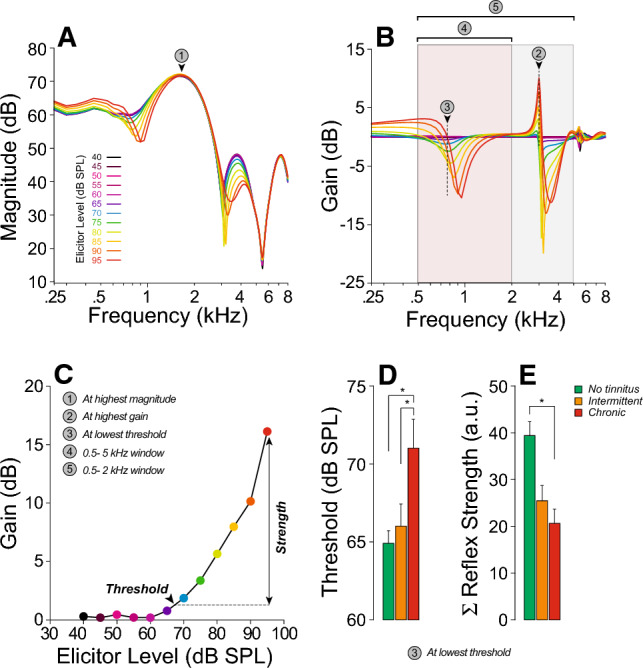
Middle-ear muscle reflex (MEMR) function is reduced in those with chronic tinnitus. (**A-C**) Exemplar data from one subject. Each curve in (**A**) is the spectrum of the ear-canal sound pressure obtained from post-elicitor clicks at one elicitor level, color-coded as shown. (**B**) Shows the corresponding spectra of the difference in sound-pressure waveforms (gain) between the pre- and post-elicitor clicks for each elicitor level. (**C**) is an example of growth function (gain vs. elicitor level) derived from (**B**). As illustrated by the circled numbers, 5 methods were used to compute MEMR thresholds and strengths (see “Materials and methods”). (**D–E**) Mean MEMR thresholds (**D**) and reflex strengths (**E**) for each group for one of the 5 methods described in (**C**). Significance of intergroup differences is indicated: *p < 0.05, **p < 0.01; ***p < 0.001.

Animal studies^80,81^ have shown that the MEMR can be a sensitive metric of CND, because low-SR fibers may be especially important in driving this sound-evoked feedback^82^. Here, MEMR strength and threshold were assessed using a pair of click probes flanking an ipsilateral noise elicitor^83^. Because the offset time constant of MEM effects is ~ 100 ms^84^, the ear-canal response to the second click is modified by lingering effects of MEM contraction on middle-ear reflectance, as illustrated in Fig. 6A. This custom wideband method yields lower reflex thresholds than those seen with clinical audiology equipment^68^. Due to the spectral complexity of the changes in ear-canal sound pressure caused by the MEM contractions (Fig. 6B), we quantify both threshold and strength of the MEMR in 5 different spectral locations (Fig. 6A,B). Mixed-effects regression analyses were used to determine the joint effect of audiometric thresholds (at standard or extended high frequencies), and tinnitus status on MEMR threshold or strength. As shown in Table 5, only tinnitus status had a significant predictive effect on MEMR metrics. Specifically, MEMR thresholds were elevated (in 4 out of 5 spectral window chosen for analysis, Table 5, Suppl. Table S6) and MEMR strength was weaker (in 2 out of 5 analysis windows, Table 5, Suppl. Table S6) in participants with chronic tinnitus.

**Table 5:** Mixed-effects regression models of stimulus level effect as a function of tinnitus status and EHFs on MEMR variables.

Predictors	At highest magnitude			At highest gain			At lowest threshold		
	Est	CI	p	Est	CI	p	Est	CI	p
MEMR thresholds									
(Intercept)	80.214	75.653 to 84.775	** < 0.001**	70.625	65.615 to 75.636	** < 0.001**	60.767	53.320 to 65.215	** < 0.001**
Standard	− 0.615	− 1.348 to 0.117	0.099	0.633	− 0.152 to 1.417	0.113	0.587	− 0.128 to 1.302	0.106
EHFs	− 0.012	− 0.227 to 0.203	0.914	0.015	− 0.224 to 0.255	0.899	0.105	− 0.104 to 0.315	0.322
Chronic	6.501	1.178 to 11.824	**0.017**	7.567	1.538 to 13.597	**0.014**	4.494	− 0.692 to 9.679	0.089
Random effects									
σ^2^	89.17			51.78			88.20		
τ_∞_	8.17_ID_			56.16_ID_			5.31_ID_		
ICC	0.08			0.52			0.06		
N	71_ID_			71_ID_			71_ID_		
Observations	104			104			104		
Marginal R^2^	0.074			0.122			0.107		
Conditional R^2^	0.151			0.579			0.158		

Predictors	500–2000 Hz window			500–5000 Hz window					
	Est.	CI	p	Est.	CI	p			
MEMR thresholds									
(Intercept)	77.439	72.624 to 82.253	**<0.001**	73.269	69.234 to 77.305	**<0.001**			
Standard	0.033	− 0.726 to 0.792	0.932	0.152	− 0.487 to 0.791	0.638			
EHFs	− 0.065	− 0.294 to 0.165	0.576	0.074	− 0.118 to 0.266	0.446			
Chronic	8.651	2.921 to 14.382	**0.003**	6.642	1.871 to 11.412	**0.007**			
Random effects									
σ^2^	57.50			43.53					
τ_∞_	42.49_ID_			24.44_ID_					
ICC	0.42			0.34					
N	71_ID_			71_ID_					
Observations	104			104					
Marginal R^2^	0.095			0.117					
Conditional R^2^	0.480			0.421					

Predictors	At highest magnitude			At highest gain			At lowest threshold		
	Est.	CI	p	Est.	CI	p	Est.	CI	p
MEMR strength									
(Intercept)	24.685	12.644 to 36.727	**< 0.001**	61.235	43.454 to 79.017	**< 0.001**	34.880	21.001 to 48.759	**< 0.001**
Standard	0.797	− 1.096 to 2.689	0.406	1.064	− 1.736 to 3.863	0.453	0.508	− 1.694 to 2.711	0.648
EHFs	− 0.071	− 0.646 to 0.503	0.806	− 0.316	− 1.164 to 0.532	0.461	− 0.052	− 0.712 to 0.608	0.875
Chronic	− 15.181	− 29.583 to − 0.780	**0.039**	− 17.001	− 38.215 to 4.213	0.115	− 17.021	− 33.398 to − 0.645	**0.042**
Random effects									
σ^2^	329.96			751.79			576.10		
τ_∞_	293.68_ID_			609.79_ID_			267.49_ID_		
ICC	0.47			0.45			0.32		
N	71_ID_			71_ID_			71_ID_		
Observations	104			104			104		
Marginal R^2^	0.055			0.042			0.049		
Conditional R^2^	0.500			0.471			0.350		

Predictors	500–2000 Hz window			500–5000 Hz window					
	Est.	CI	p	Est.	CI	p			
MEMR strength									
(Intercept)	12.983	8.767 to 17.199	**< 0.001**	119.275	85.23 to 153.32	**< 0.001**			
Standard	0.278	− 0.387 to 0.944	0.409	3.175	− 2.120 to 8.470	0.237			
EHFs	− 0.121	− 0.322 to 0.080	0.235	− 1.120	− 2.746 to 0.507	0.175			
Chronic	− 3.825	− 8.832 to 1.182	0.133	− 37.812	− 79.234 to 3.609	0.073			
Random effects									
σ^2^	46.07			2054.45					
τ_∞_	30.80_ID_			2956.94_ID_					
ICC	0.40			0.59					
N	71_ID_			71_ID_					
Observations	104			104					
Marginal R^2^	0.051			0.072					
Conditional R^2^	0.431			0.619					

*Adj.* adjusted, *Chronic* chronic tinnitus group, *CI* confidence interval, *EHFs* thresholds at extended high frequencies, *Est.* estimates, *Standard* thresholds at standard audiometric frequencies.

Significant values are in bold.

**Figure 7:**
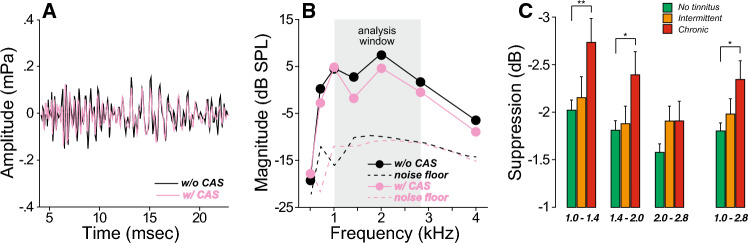
Medial olivocochlear reflex (MOCR) function is enhanced in those with chronic tinnitus. (**A**,**B**) Transient-evoked otoacoustic emissions (TEOAEs) were measured in response to clicks with or without a contralateral acoustic stimulation (CAS) consisting of a continuous broadband noise. The difference in spectral magnitude between 1 and 2.8 kHz defines the MOCR suppression. (**C**) MOCR suppression is plotted for each group of participants as a function of each frequency band or within the 1–2.8 kHz window. Significance of level effects are indicated by brackets: *p < 0.05, **p < 0.01.

The sound-evoked medial olivocochlear reflex (MOCR) is also driven by auditory nerve activity and could provide insight into the degree of CND in subjects with normal thresholds. To assess the strength of this binaural reflex, we measured the changes in transient-evoked otoacoustic emissions (TEOAEs) produced by a contralateral noise (Fig. 7). In contrast to the results with the MEMR, participants with chronic tinnitus showed *larger* MOCR-evoked suppressive effects on TEOAEs over much of the analysis window (Fig. 7C, Suppl. Table S7).

## Discussion

We estimated CND in a large sample of normal hearing participants using a test battery designed to probe different stages of the auditory system. Self-reports of chronic subjective tinnitus were associated with cochlear neural deficits, weaker MEMRs and stronger MOCRs, even when differences in sex and thresholds at standard frequencies or EHFs were accounted for.

The vast majority of our chronic tinnitus group were male, had poorer audiometric thresholds, particularly above 3 kHz, and reported more difficulties hearing in noisy environments than controls (Fig. 1). These observations are in agreement with reports showing that age-related hearing loss before age 65 is more prominent in males^33^, particularly at 4 kHz, as seen in patients with a history of noise exposure^85,86^. Also consistent with the literature^87–92^, our chronic tinnitus participants reported a history of concussion and symptoms of anxiety and/or depression more often than controls (Fig. 1). Interestingly, participants in our study with chronic tinnitus did not report more noise exposure than controls despite reporting more difficulties hearing in noisy environments, as seen in other studies^40^. The latter result is not surprising, given that the accuracy of self-reports of noise exposure is limited by the participant’s recall and greatly dependent on the number and repetitiveness of exposure episodes^93^.

### CND is associated with tinnitus

Prior histopathological studies from animal and human temporal bones have shown that the rate of cochlear neural loss greatly surpasses the rate of sensory cell loss in the aging and noise-exposed ear^24,45,74^. It is hypothesized that the loss of these neurons translates into perceptual anomalies, including tinnitus, via an induction of central gain adjustment secondary to loss of afferent input to the auditory central nervous system^51^. To test this hypothesis, we assessed the peripheral neural responses of “normal-hearing” participants with chronic tinnitus and compared them to age-matched controls using ABRs/electrocochleography, as the suprathreshold amplitude of ABR wave I is correlated with the synaptic loss when cochlear thresholds remain (or return to) normal^24,94,95^.

We found that tinnitus status was a significant predictor of cochlear neural responses, even after accounting for sex, threshold, and history of concussion, suggesting that normal hearing participants reporting chronic tinnitus have peripheral neural deficits. The fact that participants reporting intermittent tinnitus showed an intermediate phenotype further suggest that tinnitus sustainability may be dependent on the degree of peripheral neural damage.

### Recruitment of different SR groups vs. different cochlear regions to the ABR response

Many response characteristics of cochlear nerve fibers depend strongly on SR. The relationship between threshold and SR has suggested there are three distinct SR groups: low, medium and high, with progressively lower thresholds and constituting 15%, 25% and 60% of the total population, respectively^96^. Animal studies of age-related, drug-induced, and noise-induced cochlear damage suggest that the low- and medium-SR groups are more vulnerable than high-SR fibers^31,97^. Since low- and medium-SR group are also more resistant to masking, it has been hypothesized that CND may underlie the difficulties hearing noise that are so common in SNHL.

We compared the click-evoked ABR responses under several stimulus conditions to gain insight into which frequency regions and/or SR groups were contributing to the electrophysiological response differences between the chronic tinnitus vs. no tinnitus groups. The utility of click-evoked ABRs in assessing CND has been challenged given that low-SR fibers (≤ 0.5 spikes/sec) have relatively small onset responses and thus contribute less to a compound neural response^97^. However, CND affects fibers with SRs < 18 spikes/s which also includes medium-SR fibers with onset responses as robust as those of high-SR fibers^97^.

Here, the no-tinnitus group showed a significant decrement in cochlear neural response when an EHF forward masker was added, whereas the chronic tinnitus group did not (Fig. 3B1). One interpretation is that this arises from the selective loss of low- and medium-SR fibers. Since these fibers are slower to recover from a forward masker^98^, a normal ear would show a larger fractional response decrement than an ear in which there were no low-SR fibers in the EHF regions. However, the masker-probe interval in our study was only 6 ms, and the recovery time constant of even the high-SR fibers is ~ 100 msec^99^. Thus, all SR groups responding to the masker would likely still be highly fatigued at 6 ms post masker offset, and a selective loss of low-SR fibers does not provide the most likely explanation. Another possibility is that, due to their naturally high threshold, the low- and medium-SR fibers are not responding to the masker and thus are not fatigued^96,100,101^. In that case, an ear with no low- or medium-SR fibers should show a larger masking effect than a normal ear, yet, here, the chronic tinnitus group showed a smaller masking effect. Perhaps the simplest interpretation is that this arises from a reduction in the number of EHF neurons of all SR groups in the chronic tinnitus participants, and thus in their reduced fractional contribution to the unmasked ABR. The adjustment for EHF thresholds does not eliminate the intergroup difference, because primary neural degeneration would not manifest itself in the threshold measurements^24,29^.

The masker-induced latency shift that is normally seen in ABR wave I, and even more prominently in wave V, has been attributed to a shift from high- to low-SR fiber responses: the latter have longer latencies and are more resistant to maskers. Thus, normal ears show a large latency shift with increasing masker level, while neuropathic ears (without low- or medium-SR fibers) do not^102^. Here, we saw a significant reduction in the masker-induced latency shift in the chronic tinnitus ears, which could therefore reflect a selective loss of low/medium-SR fibers. However, a recent single-fiber study of auditory-nerve responses to fixed-frequency tone pips suggested that the masker-induced latency shift in ABR peaks likely arises from a shift from high-SR fibers tuned to the tone-pip frequency to high-SR fibers in more basal cochlear regions responding (with longer latencies) at the low-frequency “tail” of their tuning curves^103^. Thus, the lack of masker induced latency shift seen here could also be due to the relative paucity of fibers in the EHF region, regardless of their SR.

The same basic confound applies to the interpretation of the intergroup differences in level effect shown in Fig. 5. As the click level is increased, the response amplitude normally increases both by recruitment of high-threshold, low-SR fibers and by recruitment of fibers from all SR groups in the EHF regions^96^. These high-frequency fibers are more difficult to stimulate because (1) their absolute thresholds are higher than those in the standard frequency range and (2) the frequency response of our acoustic system (the ER-3A) rolls off above 4 kHz and thus the click spectrum contains relatively less energy at EHFs than at standard frequencies^44^ (see Suppl. Fig. S1). Although a selective loss of low-SR fibers would decrease the level-dependent enhancement of ABR amplitudes, so would the loss of neurons of all SRs in the EHF region.

We also probed the contributions of low- and medium-SR fibers to the EcochG by increasing click rate from 9.1 to 40.1 Hz, given that (1) neural potentials adapt^104^ at high presentation rates and (2) low- and medium-SR fibers are more fatigued by increasing stimulus rate than their high-SR counterparts^98,99,101^. As shown in Fig. 4B_1_,B_2_, although the AP^*^ amplitude was reduced at high rates in both groups, the chronic tinnitus group showed less of a rate effect than the control group. In contrast to the other stimulus manipulations, this one is not subject to the EHF confound and would be consistent with a selective loss of low/medium-SR fibers in the chronic tinnitus group.

Altogether, the EcochG results strongly suggests a loss of cochlear neurons in the chronic tinnitus group and are consistent with the low- and medium-SR fiber population being over-represented in that missing neuron pool.

### Auditory efferent reflexes

There are two efferent, sound-evoked neuronal feedback pathways to the auditory periphery: the MEMR and MOCR^105^. Both circuits comprise a three-neuron arc starting with cochlear nerve projections to the cochlear nucleus. For both reflexes it has been suggested that the low-SR fibers might be over-represented in the afferent limb^59,60^. For the MEMR, cochlear nucleus neurons project to facial motoneurons, which in turn project to the stapedius muscle, but the cochlear nucleus subtype has been poorly characterized^105^. For the MOCR^106^, a class of multipolar cells in the anteroventral and posteroventral cochlear nucleus project to MOC neurons in the superior olivary complex^106^, which in turn project to cochlear outer hair cells, thus controlling cochlear gain^106^.

We found that chronic tinnitus was associated with weaker MEMR strengths and higher MEMR thresholds (Fig. 6). The significance of the intergroup difference remained after adjusting for threshold and thus cannot be attributed to outer hair cell dysfunction, either in the standard or EHF ranges. Our results are in line with animal studies showing that CND, as measured histopathologically, correlates with measures of the MEMR^80,81^. In humans, an MEMR study using tonal elicitors, as performed in clinical settings, didn’t find a statistically significant association of tinnitus with the MEMR *threshold*^107^. On the other hand, when the suprathreshold growth of the MEMR *strength* was assessed with a more sensitive metric^68^ similar to our protocol, those with tinnitus had significantly weaker MEMRs than those without^52^.

In contrast to the MEMR effects, but in line with a number of prior studies^61,108,109^, we observed greater MOCR effects in the chronic tinnitus group. This discrepant behavior of the two reflexes could arise from differences in the extent to which each is integrated with other central auditory circuits. Indeed, MOC neurons have a rich descending projection from the inferior colliculus^110^, which in turn integrates many ascending and descending projections, including from the dorsal cochlear nucleus, where robust hypersensitivity arises after peripheral damage^110^. Increased MOCR effects on otoacoustic emissions are also observed in studies involving visual and auditory attention tasks^111–113^. The stapedius motoneurons, on the other hand, may not be as richly interconnected with other major auditory centers^114^. Therefore, one possible interpretation of these results is that MOC efferents in tinnitus participants receive excitatory inputs from higher centers of the auditory pathways due to central gain.

### Central gain and tinnitus

As shown here, and in other human studies^50,51,115^, participants with tinnitus had reduced wave I (AP^*^) amplitudes but enhanced wave III/I amplitude ratios (reduced N_1_^*^/N_3_^*^ ratios: Fig. 2), suggesting a gain boost between the response of the cochlear nerve and the inferior colliculus. In our study, these signs of central hyperactivity were also present in wave II but not apparent in wave V. Animal studies^116,117^ suggest that wave II and III are dominated by activity in pathways originating in the ventral cochlear nucleus, with the globular cell and spherical cell pathways respectively, but there is also evidence that wave II has contributions from the auditory nerve^118^. Increased excitability of the cochlear nucleus pathways has been shown in guinea pigs following acoustic trauma causing permanent threshold shifts^119^, and increased excitability in the inferior colliculus and cortex have been seen in mice following a near-complete cochlear denervation^23,43^.

**Figure 8:**
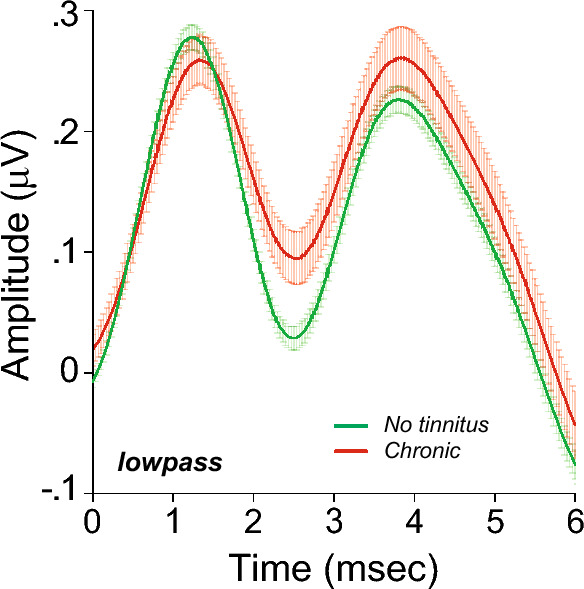
Increase in central gain is also detected in low passed ABR waveforms: Averaged click-evoked ECochG (± SEMs) obtained from each group were band-pass filtered (3–470 Hz). Baseline is defined as the mean pre-onset amplitude (− 2 to 0 ms).

While our results on the later ABR waves are largely similar to that seen in previous reports^50,51,115,120,121^, the lack of intergroup differences in Wave V/I amplitude ratio is remarkable. Our passband filtering of the EcochG waveforms and the use of baseline-to-peak measure, rather than a trough-to-peak measure for wave V amplitude, may contribute to this difference. As illustrated in Fig. 8, intergroup comparison of the low-pass component of ABRs indicates an enhancement of the waveform generators at post-Wave I latencies (p < 0.001), consistent with hyperactive generators in the central auditory pathways of tinnitus patients.

It is also important to note that CND, irrespective of OHC loss, is unlikely to be sufficient to evoke the central changes necessary for the development of a tinnitus percept, as many patients with sensorineural hearing loss do not report tinnitus. Beside the central gain observed as the result of cochlear damage^23,43^, additional failures in central auditory pathways (e.g. failure of the thalamic gating^16^) may be necessary for the development of an anomalous perception.

## Conclusion

This study clarifies the association between biomarkers of peripheral neural deficits with tinnitus and is consistent with the idea that CND may serve as a peripheral trigger for excess central gain^43,122,123^. Future psychophysical measures of tinnitus and sound-level intolerance may help interpret the pathology underlying the changes in physiological responses including at higher stages of the auditory system. They may also clarify the role of CND in the development and maintenance of central hyperactivity and the engagement of autonomically driven changes in the affective responses to sound. In a noise-damaged mouse model, neurotrophin overexpression via gene therapy or supplementation via local delivery can elicit regeneration of ANF connections with IHCs^124,125^. Developing diagnostic assays of CND in humans and clarifying its link to the genesis and/or maintenance of the tinnitus percept is therefore key to identify candidates for future therapeutics and to track the efficacy of any treatments designed to rebuild a damaged inner ear and perhaps reverse the tinnitus percept.

### Supplementary Information

Supplementary Figure 1.

Supplementary Tables.

## Data Availability

All relevant data are within the paper and its Supporting Information files. Raw data that support the findings of this study are available from the corresponding author, upon reasonable request.
