# Supplementary figures and images for: Evidence of cochlear neural degeneration in normal-hearing subjects with tinnitus
Source: Sci Rep. 2023 Nov 30;13:19870. doi: 10.1038/s41598-023-46741-5 (PMC10689483; doi:10.1038/s41598-023-46741-5)

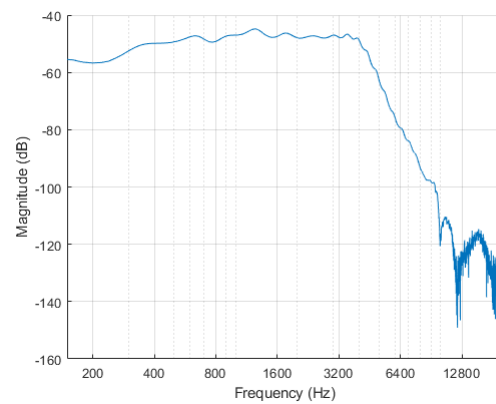

Supplement: Supplementary file 1 — Supplementary Figure 1. [file 41598_2023_46741_MOESM1_ESM.pdf]
