# Supplementary material for: Evidence of cochlear neural degeneration in normal-hearing subjects with tinnitus
Source: Sci Rep. 2023 Nov 30;13:19870. doi: 10.1038/s41598-023-46741-5 (PMC10689483; doi:10.1038/s41598-023-46741-5)
Supplement: Supplementary file 2 — Supplementary Tables. [file 41598_2023_46741_MOESM2_ESM.pdf]

Supplemental tables

Predictors	*Est.*	*CI*	*p*
(intercept)	5.49	3.95 – 7.03	**<0.001**
no tinnitus	0.48	-1.07 – 2.04	0.540
chronic	3.34	0.93 – 5.74	**0.010**
Observations	8230		
Marginal R^2 / Conditional R^2	0.011 / N/A		

Supplemental Table 1: Analysis of audiometric threshold differences using mixed-effects regression. The group of participants with intermittent tinnitus was set as baseline. Est.: Estimates.

	no tinnitus vs. chronic		*no tinnitus vs. intermittent*		*intermittent vs. chronic*	
Variable	*t*	*p*	*t*	*p*	*t*	*p*
AP^* amplitude	-3.223	**0.002**	-2.043	**0.043**	1.256	0.214
N_1^*/N_2^*	-3.203	**0.002**	-2.455	**0.015**	1.791	0.079
N_1^*/N_3^*	-2.102	**0.037**	-2.008	**0.046**	0.469	0.641
N_1^*/N_5^*	-0.500	0.618	-1.932	0.055	-1.068	0.290
N_1^* latency	0.565	0.573	-0.082	0.935	-0.523	0.604
N_2^* latency	0.364	0.716	1.023	0.308	0.478	0.635
N_3^* latency	0.408	0.684	0.753	0.452	0.203	0.840
N_5^* latency	1.530	0.128	0.052	0.959	-1.240	0.220
N_5^*- N_1^* latency	1.396	0.165	0.167	0.868	-0.833	0.409

Supplemental Table 2: Pairwise comparisons between groups using mixed-effects model with groups in fixed-effects and participants in random effects.

	no tinnitus		*chronic*	
	no masker vs. masker		*no masker vs. masker*	
Variable	*t*	*p*	*t*	*p*
AP^* amplitude	8.179	**<0.001**	1.677	0.098
N_1^*/N_2^*	4.707	**<0.001**	1.900	0.062
N_1^*/N_3^*	4.412	**<0.001**	0.455	0.650
N_1^*/N_5^*	2.665	**0.008**	2.849	**0.006**
N_1^* latency	-4.324	**<0.001**	-1.170	0.246
N_2^* latency	-3.495	**0.001**	0.376	0.708
N_3^* latency	-6.666	**<0.001**	-0.304	0.762
N_5^* latency	-7.536	**<0.001**	0.133	0.894
N_5^*- N_1^* latency	-4.596	**<0.001**	0.542	0.589

Supplemental Table 3: Pairwise comparisons between unmasked vs. masked conditions using mixed-effects model with masker effects in fixed effects and participants in random effects.

	no tinnitus		*chronic*	
	9.1 vs. 40.1 Hz		*9.1 vs. 40.1 Hz*	
Variable	*t*	*p*	*t*	*p*
AP^* amplitude	20.504	**<0.001**	7.551	**<0.001**
N_1^*/N_2^*	1.361	0.174	-0.108	0.915
N_1^*/N_3^*	2.428	**0.016**	0.842	0.403
N_1^*/N_5^*	4.114	**<0.001**	4.413	**<0.001**
N_1^* latency	-7.211	**<0.001**	-3.352	**0.001**
N_2^* latency	-7.705	**<0.001**	-3.720	**<0.001**
N_3^* latency	-12.527	**<0.001**	-5.934	**<0.001**
N_5^* latency	-14.158	**<0.001**	-6.708	**<0.001**
N_5^*- N_1^* latency	-11.308	**<0.001**	-2.397	**0.019**

Supplemental Table 4: Pairwise comparisons between slow (9.1 Hz) vs. fast rate (40.1 Hz) conditions using mixed-effects model with rate effects in fixed effects and participants in random effects.

	no tinnitus		*chronic*	
	110 vs. 125 dBpSPL		*110 vs. 125 dBpSPL*	
Variable	*t*	*p*	*t*	*p*
AP^* amplitude	-7.158	**<0.001**	-1.660	0.108
N_1^*/N_2^*	2.667	**0.009**	1.720	0.096
N_1^*/N_3^*	-0.106	0.916	1.427	0.164
N_1^*/N_5^*	-2.639	**0.009**	0.489	0.629
N_1^* latency	9.491	**<0.001**	6.448	**<0.001**
N_2^* latency	11.013	**<0.001**	5.894	**<0.001**
N_3^* latency	8.523	**<0.001**	3.716	**0.001**
N_5^* latency	6.960	**<0.001**	2.991	**0.006**
N_5^*- N_1^* latency	-0.229	0.819	-0.329	0.745

Supplemental Table 5: Pairwise comparisons between stimulus presentation levels (110 vs. 125 dB pSPL) using mixed-effects model with level effects in fixed effects and participants in random effects.

MEMR threshold

	control vs. chronic		*control vs. intermittent*		*intermittent vs. chronic*	
Method	*t*	*p*	*t*	*p*	*t*	*p*
At highest mag.	2.096	**0.040**	-1.876	0.064	-2.965	**0.006**
At highest gain	3.011	**0.004**	0.475	0.636	-2.643	**0.013**
.5-5 kHz window	3.246	**0.002**	-0.272	0.786	-3.853	**0.001**
At lowest thr.	2.523	**0.013**	-0.285	0.776	-2.438	**0.020**
.5-2 kHz window	2.989	**0.004**	1.095	0.277	-2.472	**0.020**

MEMR strength

	control vs. chronic		*control vs. intermittent*		*intermittent vs. chronic*	
Variable	*t*	*p*	*t*	*p*	*t*	*p*
At highest mag.	-2.087	**0.040**	-0.559	0.578	2.073	**0.046**
At highest gain	-1.722	0.089	-0.881	0.381	1.045	0.304

.5-5 kHz window	-2.018	**0.047**	-0.366	0.715	2.162	**0.038**
At lowest thr.	-2.125	**0.038**	-1.190	0.238	1.220	0.232
.5-2 kHz window	-1.752	0.084	-1.886	0.063	0.231	0.819

Supplemental Table 6: Pairwise comparisons between groups using mixed-effects model with groups in fixed-effects and participants in random effects.

	control vs. chronic		*control vs. intermittent*		*intermittent vs. chronic*	
	t	*p*	*t*	*p*	*t*	*p*
1.0 – 1.4	2.618	**0.009**	0.598	0.550	-1.622	0.107
1.4 – 2.0	2.294	**0.023**	0.345	0.730	-1.644	0.103
2.0 – 2.8	1.479	0.141	1.892	0.059	-0.005	0.996
1.0 – 2.8	2.532	**0.012**	1.033	0.302	-1.364	0.175

Supplemental Table 7: Pairwise comparisons of MOCR strength between groups.
